# Genome sequencing of *Pseudomonas aeruginosa* strain M2 illuminates traits of an opportunistic pathogen of burn wounds

**DOI:** 10.1093/g3journal/jkac073

**Published:** 2022-03-28

**Authors:** Victoria I Verhoeve, Jerod A Brammer, Timothy P Driscoll, Adrienne R Kambouris, David A Rasko, Alan S Cross, Joseph J Gillespie

**Affiliations:** 1 Department of Microbiology and Immunology, University of Maryland School of Medicine, Baltimore, MD 21201, USA; 2 Department of Biology, West Virginia University, Morgantown, WV 26506, USA; 3 Institute for Genome Sciences, University of Maryland School of Medicine, Baltimore, MD 21201, USA; 4 Center for Vaccine Development, Institute for Global Health, University of Maryland School of Medicine, Baltimore, MD 21201, USA

**Keywords:** *Pseudomonad aeruginosa*, PA M2, PA14, phylogenomics, burn model

## Abstract

*Pseudomonas aeruginosa* is a Gram-negative nosocomial pathogen and one of the most prevalent organisms isolated from burn wounds worldwide. *Pseudomonas aeruginosa* strain M2 (O5 serotype, type B flagella) is utilized for examining the murine model associated with burns. *Pseudomonas aeruginosa* M2 is similar in lethality to common laboratory *P. aeruginosa* strains when infecting CD-1 mice. Conversely, we recently showed that, relative to these strains, *P. aeruginosa* M2-infected mice are more susceptible to sepsis and demonstrate a 6-log reduction in LD_50_ from subcutaneous infection at the infection site directly after 10% total body surface area burn. To better understand this striking phenotypic difference from other *P. aeruginosa* strains employed in burn models, we sequenced the *P. aeruginosa* M2 genome. A total of 4,136,641 read pairs were obtained, providing an average genome coverage of 97.5X; subsequent assembly yielded a draft genome with 187 contigs comprising 6,360,304 bp with a G + C content of 66.45%. Genome-based phylogeny estimation of 92 *P. aeruginosa* strains placed *P. aeruginosa* M2 with *P. aeruginosa*-12-4-4(59), a nonairway clinical strain isolated from the blood culture of a burn patient. Phylogenomic analyses identified genes shared between *P. aeruginosa* M2 and *P. aeruginosa* 14, another strain exhibiting increased lethality in thermal tissues, as well as *P. aeruginosa* M2 unique genes with diverse functions like degradation of toxic aromatic compounds, iron scavenging, swarming motility and biofilm formation, defense against invasive DNA, and host assault. Predicted lateral gene transfers illuminate proteins heretofore uncharacterized for roles in *P. aeruginosa* biology. Our work yields a rich resource for assessing *P. aeruginosa* genes required for increased lethality in burn tissue seroma.

## Introduction 

*Pseudomonas aeruginosa* is a ubiquitous rod-shaped Gram-negative bacterium. *Pseudomonas aeruginosa* is commonly found on indoor surfaces where people live and work, yet so thrives in diverse environmental niches such as soil, water, and plants (De Abreu *et al.* 2014; Rutherford *et al.* 2018). *Pseudomonas aeruginosa* also colonizes intestinal tracts of immunocompromised humans (Griffith *et al.* 1989) and is a major multidrug-resistant opportunistic pathogen identified clinically worldwide. In the United States, *P. aeruginosa* caused 32,600 infections in hospitalized patients and resulted in 2,700 deaths in 2019 (2019 AR Threats Report CDC).

Burn patients are susceptible to infection by *P. aeruginosa*, which is the most commonly identified Gram-negative bacterium isolated from burn wounds (Azzopardi *et al.* 2014; Norbury *et al.* 2016). Various animal models (e.g. mouse, rat, and pig) are employed to study how *P. aeruginosa* causes pathology in burn patients, with different methods (i.e. scalds, contact burns, and flame burns) used to simulate burns of varying type and severity (Abdullahi *et al.* 2014). Researchers typically use common laboratory *P. aeruginosa* strains (e.g. PAO1 and PA14) to study postburn pathological effects (Rumbaugh *et al.* 1999; Barnea *et al.* 2006; Dzvova *et al.* 2018; Elmassry *et al.* 2020); however, little is known about how *P. aeruginosa* strains differentially infect burn wounds and vary in virulence.

Previously, Stieritz and Holder developed a nonlethal ethanol-based flame burn in mice that was initially used to assess the increased susceptibility to subsequent bacterial infections (Stieritz and Holder 1975). They observed a 6-log reduction in LD_50_ for a subcutaneous infection with *P. aeruginosa* strain M2 (a mouse isolate) from 1.3 × 10^6^ to <10 CFU. Recently, our laboratory further characterized this procedure using PAO1 and PAO10 (Brammer *et al.* 2021). While displaying similar lethality to PAO1 and PAO10 in unburned mice, PA M2 exhibited an LD_50_ 6-logs lower than PAO1 and PAO10 in burned mice. In order to identify genetic factors that possibly underpin increased *P. aeruginosa* M2 virulence in burned mice, we sequenced this strain’s genome and performed comparative genomics to identify *P. aeruginosa* M2 unique characteristics. This identified numerous genes for the future assessment of disease severity of *P. aeruginosa*-infected burn patients.

## Materials and methods

### Strain origin, cultivation, and electron microscopy

*Pseudomonas aeruginosa* M2, originally isolated from the intestine of a CF-1 mouse (Stieritz and Holder 1975), was previously obtained from Dr. Alan Holder and has been used in our laboratories for decades. To visualize *P. aeruginosa* M2, bacteria were grown in Hy-soy broth at 37°C overnight with shaking at 220 rpm. Colonies were re-suspended in water, applied onto glow-discharged 400 mesh carbon-coated copper grids and negatively stained with freshly prepared 1% uranyl acetate (wt/vol). Grids were air-dried and examined in a transmission electron microscope (Tecnai T12, Thermo Scientific) at an operating voltage of 80 kV. Digital images were acquired using an AMT bottom mount CCD camera and AMT600 software.

### Genome sequencing, assembly, and annotation

Purified genomic DNA was obtained from 1 ml of overnight *P. aeruginosa* M2 culture using the Promega Wizard Genomic DNA purification Kit (Promega, Fitchburg, WI, USA) according to manufacturer’s specifications. Sequencing was performed by the Microbial Genome Sequencing Center (https://www.migscenter.com/). Library prep was conducted using a modified version of Nextera DNA kits with no size selection and sequenced on a NextSeq 550 (Baym *et al.* 2015). Quality control and adapter trimmer was performed with bcl2fastq v2.20 (https://support.illumina.com/sequencing/sequencing_software/bcl2fastq-conversion-software.html). Assembly was performed with SPAdes version: 3.13.0 (Nurk *et al.* 2013) and only contigs >2,000 bp were included. Gene prediction and assembly annotation were performed with RAST (Aziz *et al.* 2008). Default parameters were used for all software unless otherwise specified.

### Phylogeny estimation

Single nucleotide polymorphisms (SNPs) were identified in *P. aeruginosa* M2 and 90 *P. aeruginosa* genomes using the Northern Arizona SNP Pipeline (NASP) with default parameters (Sahl *et al.* 2016) and PAO1 as a reference (NC_002516_2). SNPs were filtered to remove sites in regions duplicated in PAO1, sites with missing data, and monomorphic sites. The nonduplicate SNPs present in all genomes were concatenated into a dataset used for phylogeny estimation with IQ-TREE v.1.6.12 (Nguyen *et al.* 2015). A maximum-likelihood phylogeny was inferred using the best-fit substitution model (GTR+F+ASC+R5) determined by ModelFinder (Kalyaanamoorthy *et al.* 2017) and with ascertainment bias correction. Bootstrap support was determined using ultrafast bootstrap approximation run with 1,000 replicates and the bnni option to reduce overestimating support (Hoang *et al.* 2018).

### Phylogenomics analyses

*Pseudomonas aeruginosa* M2, PAO1, and PA14 (ASWV01000001.1) proteins were used in an LS-BSR analysis (Sahl *et al.* 2014). Subjects from “all-against-all” blastp searches were ranked by normalized scores (BLAST score of the best hit in the query genome divided by the BLAST score of the gene of interest to itself). A BSR score of 0.8 was selected as a threshold (∼80% aa identity over 80% length of the interrogated peptide), with lower scores capturing divergent proteins shared between genomes (e.g. a BSR score ≤ 0.4 is less than 30% identify over 30% of the peptide) or proteins unique to each genome (BSR score of zero). Proteins either shared by PA14 and *P. aeruginosa* M2 or unique to *P. aeruginosa* M2 were then separated into “singly occurring” or “clustered” on assembled contigs and manually assigned to one of 12 functional categories based on predicted annotations. All proteins were evaluated for pseudogenization (proteins comprising less than 40% of most other *P. aeruginosa* or other bacterial homologs, as well as evidence for fragmentation) and spurious CDS (short with zero or minimal blastp hits to the NCBI nr database). All hypothetical proteins were further evaluated with the NCBI Conserved Domains Database (Lu *et al.* 2020) and SMART (Letunic and Bork 2017) following previous approaches (Gillespie *et al.* 2018).

The *P. aeruginosa* M2 genome was further analyzed with HaloBLAST, a combinatorial blastp-based approach for interrogating proteins for later gene transfer (LGT) (Driscoll *et al.* 2013). All *P. aeruginosa* M2 proteins were used as queries in blastp searches against 5 distinct taxonomic databases: (1) “Pseudomonas excluding *Pseudomonas aeruginosa*,” (2) “Pseudomonadaceae excluding Pseudomonas,” (3) “Pseudomonadales excluding Pseudomonadaceae,” (4) “Gammaproteobacteria excluding Pseudomonadales,” and (5) “Bacteria excluding Gammaproteobacteria.” The top 200 subjects from each search were merged and ranked by *Sm* score, a comparative sequence similarity score designed to de-emphasize highly significant matches to short stretches of query (i.e. conserved domains) in favor of longer stretches of similarity (Driscoll *et al.* 2013). The “halo” or database having all or the majority of subjects was then assigned to each query protein, with “non-Pseudomonas” assignments considered evidence for LGT.

## Results and discussion

**Fig. 1.**
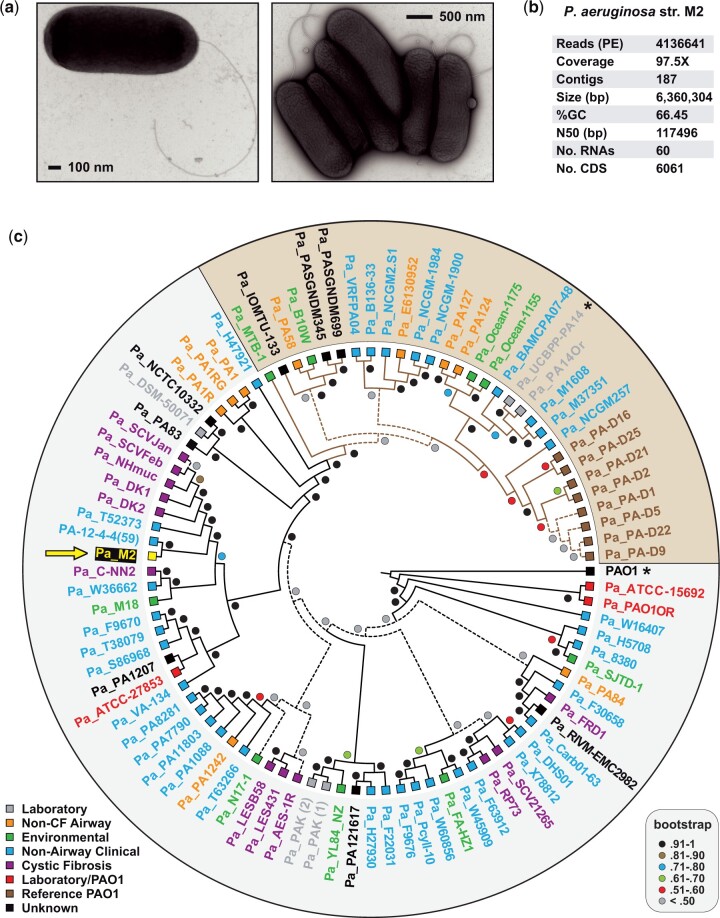
Characteristics of *P. aeruginosa* M2. a) Electron microscopy showing a single bacterium with unipolar flagella (left) and a group of bacteria tightly clustered (right). b) Genome sequencing and assembly statistics for *P. aeruginosa* M2. c) Phylogeny estimation for *P. aeruginosa* M2 and 91 other *P. aeruginosa* strains. Tree is based on SNP divergence and computed using NASP (Sahl *et al.* 2016). Branch support is from 1,000 bootstrap pseudoreplications. *Pseudomonas aeruginosa* M2 is noted with the arrow. Shading, divergent clade. Asterisks denote strains PAO1 and PA14 used in the LS-BSR analysis (Fig. 2a). A phylogram is shown in Supplementary Fig. 1 with NCBI accession numbers for all genomes.

Visualization of *P. aeruginosa* M2 (O5 serotype, type B flagella) using electron microscopy revealed typical *P. aeruginosa* phenotypic characteristics, including long unipolar flagella and bacterial clustering (Fig. 1a). Genome sequencing of *P. aeruginosa* M2 yielded a total of 4136641 read pairs at an average genome coverage of 97.5X (Fig. 1b). A draft genome assembly was generated containing 187 contigs with an N50 of 117,496 bp. The assembly characteristics, including genome sequence length (6,360,304 bp), %GC (66.45), and number of coding sequences (6,061) are all typical of other sequenced *P. aeruginosa* genomes (Winsor *et al.* 2016).

To better understand its evolution within *P. aeruginosa*, we estimated a genome-based phylogeny for *P. aeruginosa* M2 and 91 additional *P. aeruginosa* strains (Fig. 1c). *Pseudomonas aeruginosa* M2 grouped with PA-12-4-4(59) (NZ_CP013696.1), a nonairway clinical strain isolated from the blood culture of a burn patient (Karna *et al.* 2016). This clade occurs with the majority of other selected *P. aeruginosa* strains (67%) that are highly divergent from a minority of the analyzed *P. aeruginosa* genomes, including PA14 (33%; brown shading; Supplementary Fig. 1). Despite this, strain characteristics (source, patient information, geography, etc.) segregate sporadically across the phylogeny.

**Fig. 2.**
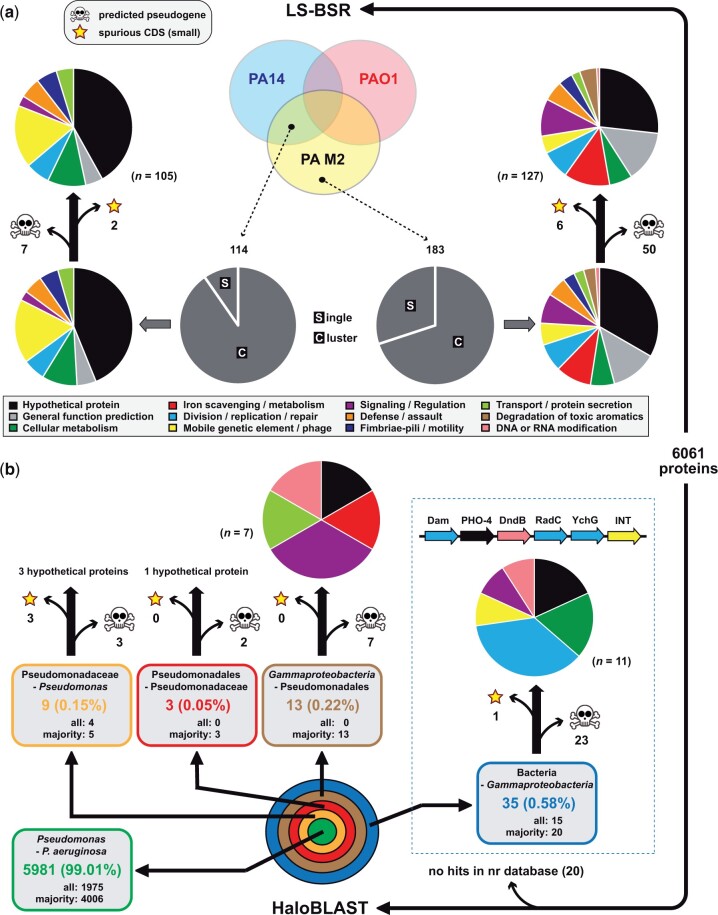
Phylogenomics analysis of *P. aeruginosa* M2. a) LS-BSR analysis for *P. aeruginosa* M2 and strains PAO1 and PA14. Venn diagram illustrates the 114 proteins shared by PA14 and *P. aeruginosa* M2 and the 183 *P. aeruginosa* M2-defining proteins either absent in the other strains or highly divergent from PAO1 and PA14 counterparts (BSR score ≤ 0.4 equating to less than 30% identity over 30% of the *P. aeruginosa* M2 protein). Pie charts show predicted functional categories for single or clustered proteins before and after manual evaluations for pseudogenes and spurious CDS. b) HaloBLAST analysis for all *P. aeruginosa* M2 proteins. Concentric halos depict hierarchical taxonomic databases increasing in divergence from the center. Ellipses capture the results from merging the top 10 scoring subjects from each database search; e.g. in the “Pseudomonadaceae minus Pseudomonas” box, 4 query *P. aeruginosa* M2 proteins had all top 10 hits to this database, whereas another 5 queries had the majority of their top 10 hits to this database. The dashed box encloses the predicted mobile modification system involved in 7-deazaguanine (or derivatives) insertion into DNA: Dam, D12 class N6 adenine-specific DNA methyltransferase (pfam02086); PHO-4, phosphate transporter (pfam01384); DmdB, DNA-sulfur modification protein (pfam14072); RadC, DNA repair and recombination protein (COG2003); YchG, predicted nuclease of restriction endonuclease-like (RecB) superfamily (COG4804); INT, integron-like integrase/recombinase (cd00796). NOTE: 5 diverse *P. aeruginosa* genomes were found to carry these genes in a similar contiguous fashion and at 100% aa identity: str. PABL012 (blood from patient in Chicago, Illinois, AXR28215), str. PSA00040 (urine from patient at University of Pittsburgh Medical Center, MBG5741906), str. PSA00018 (blood from patient at University of Pittsburgh Medical Center, MBG5887200), str. UMB1204 (urine, Maywood, IL, MWW64768), and str. T2101 (adult male sputum, Bangkok, Thailand, QGQ03306). All corresponding information for proteins from LS-BSR and HaloBLAST analyses are provided in Supplementary Table 1.

We used 2 phylogenomics-based approaches to identify genes defining *P. aeruginosa*’s unique phenotype in the seroma layer of burns (Fig. 2). First, we employed a Large Scale BLAST Score Ratio (LS-BSR) analysis (Sahl *et al.* 2014) between *P. aeruginosa* M2 and one strain lacking (PAO1) and another possessing (PA14) decreased lethality in burn models (Fig. 2a). Proteins shared by strains exhibiting a lower LD_50_ in seroma tissues (PA14 and *P. aeruginosa* M2) and proteins defining *P. aeruginosa* M2 alone were then separated into “singly occurring” or “clustered” (i.e. tandemly arrayed) on assembled contigs, assigned to one of 12 functional categories based on predicted annotations, and scanned for pseudogenization and spurious CDS.

Proteins shared by PA14 and *P. aeruginosa* M2 (*n *=* *114) were largely clustered on contigs (90%) and included few pseudogenes or spurious CDS (*n *=* *9). In contrast, proteins defining *P. aeruginosa* M2 alone (*n *=* *183) had fewer clustered on contigs (70%) and contained many more pseudogenes or spurious CDS (*n *=* *56). A greater proportion of mobile genetic elements (MGEs) was found in proteins shared by PA14 and *P. aeruginosa* M2 (18%) relative to proteins defining *P. aeruginosa* M2 alone (6%); e.g. 12 proteins shared by PA14 and *P. aeruginosa* M2 were annotated as components of PFGI-like integrative conjugative elements (Mavrodi *et al.* 2009) vs only one specific to *P. aeruginosa* M2. Despite sharing a reduced LD_50_ in seroma tissue, the PA14 heparinase gene (*hepP*) previously demonstrated to be critical for pathogenesis in burn wound infection (Dzvova *et al.* 2018) was not detected in the *P. aeruginosa* M2 genome. This implies that multiple genetic factors underpin greater pathogenesis of some *P. aeruginosa* strains in tissues following thermal insult. Nonetheless, despite most having homologs in other *P. aeruginosa* genomes, these proteins shared between PA14 and *P. aeruginosa* M2 warrant testing for functions associated with *P. aeruginosa* aggressiveness in endothelial seroma.

Of the 183 proteins defining *P. aeruginosa* M2, the majority (75%) are absent from PAO1 and PA14 genomes (*n *=* *61) or highly divergent from PAO1 and PA14 counterparts (*n *=* *76). Accordingly, these proteins serve as potential factors underlying *P. aeruginosa* M2’s pathogenesis in endothelial seroma. The most noticeable differences at the functional category level relative to the proteins shared by PA14 and *P. aeruginosa* M2 involve degradation of toxic aromatics, signaling and regulation, as well as iron utilization (Fig. 2a;Supplementary Fig. 2). Regarding the last, 10 proteins are predicted to synthesize pyoverdines, siderophores with characterized roles in virulence and biofilm formation in pseudomonads (Lamont *et al.* 2002; Banin *et al.* 2005). Six additional proteins are involved in iron scavenging, with 2 grouped in a pyoverdine-encoding gene cluster, raising the possibility that *P. aeruginosa* M2 sequesters and internalizes host iron differently than strains PAO1 and PA14.

Several functional categories are enriched within clustered genes (Supplementary Fig. 2). Four type I fimbriae proteins are clustered with 4 proteins involved in sensing stimuli, signaling, or transcriptional regulation, with the overall profile of this cluster possibly comprising an RcsCDB signal transduction system that regulates swarming motility (Wang *et al.* 2007). Proteins with general functions in DNA replication, cell division, and DNA repair were found to be enriched in 3 *P. aeruginosa* M2-defining clusters. Overall profiles for these clusters suggest MGEs carrying genes for DNA modification. One cluster encodes a bacterial PIWI module, a restriction endonuclease fold enzyme and a DinG family helicase, which collectively comprise an RNA-dependent restriction modification (RM) system thought to restrain transcription of invading DNA (i.e. phages, plasmids, or conjugative transposons) by utilizing RNA guides (Burroughs *et al.* 2013). A second cluster is also predicted to function in defense, as it carries genes characteristic of BacteRiophage EXclusion (BREX) systems (Chaudhary 2018) including the BREX-3 system phosphatase PglZ, DNA helicases, a DNA methylase and sirtuin-like domain that likely regulates the element. The third cluster carries genes encoding DNA repair (RadC), methylation (Dam) and phosphorothioation enzymes (DndB), a RecB-like endonuclease, and an integron-like integrase/recombinase (NCBI conserved domain cd00796) possibly constituting an RM system that inserts 7-deazaguanine derivatives in DNA (Thiaville *et al.* 2016). These 3 clusters collectively illustrate *P. aeruginosa* M2’s acquisition of MGEs encoding mechanisms for defense against phage and other invasive DNA.

The largest cluster (*n *=* *14) encodes proteins with diverse functions (e.g. metabolism, drug efflux, and regulation) but most importantly a putative sialidase with several bacterial neuraminidase repeats. A second probable neuraminidase (pfam15892: BNR_4) is found in a 3 gene cluster and was detected in only a few other *P. aeruginosa* genomes. These proteins, along with 2 nonclustered proteins predicted as a C80 peptidase (cd20500) and a BapA prefix-like domain-containing protein with many Ig domains and T1SS RTX-like signal, are candidate secreted effectors worthy of investigating for roles in *P. aeruginosa* M2 seroma layer colonization.

Our second phylogenomics-based approach entailed predicting LGT between *P. aeruginosa* M2 and more distant bacteria. Depending on the set of analyzed genomes, the *P. aeruginosa* accessory genome can comprise as much as 21% of total genes and is rich in genes with diverse functions, duplications, and MGEs (Kung *et al.* 2010; Pohl *et al.* 2014). Aside from environmental strains, clinical isolates also harbor diverse genes of the *P. aeruginosa* accessory genome (Shen *et al.* 2006). Accordingly, we analyzed all *P. aeruginosa* M2 proteins with HaloBLAST, a method that determines the predominant sequence similarity across restricted hierarchical taxonomic databases (Driscoll *et al.* 2013). This approach determined that 99% of *P. aeruginosa* M2 proteins have widespread distribution in other *Pseudomonas* genomes (Fig. 2b). For the remaining proteins (*n *=* *60), blastp searches determined 39 are either pseudogenes or short spurious CDS (Supplementary Table 1). The degree of pseudogenization increases linearly for proteins predicted to be acquired from nonpseudomonad bacteria, indicating that most LGTs from distant microbes (particularly *Neisseria* spp.) are disintegrating from the *P. aeruginosa* M2 genome.

Eliminating pseudogenes from the small pool of LGTs from more distant microbes allowed for evaluating other LGTs that have been selected for in the *P. aeruginosa* M2 genome. For these proteins (*n *=* *21), 12 were also identified in the LS-BSR analysis (Supplementary Table 1) supporting their uniqueness in *P. aeruginosa* M2 relative to strains PAO1 and PA14. The other 9 proteins are hypothetical (*n *=* *3) or have predicted functions (transport, iron acquisition, DNA modification, transcriptional regulation, or metabolism) and are either not detected in most *P. aeruginosa* genomes or have stronger similarity in distantly related bacteria. Their relevance to the biology of *P. aeruginosa* M2 remains to be determined.

Finally, the abovementioned RM system involved in 7-deazaguanine (or derivatives) insertion into DNA was also detected using HaloBLAST (inset in Fig. 2b), with the majority of similar sequences occurring in genomes from nongammaproteobacterial species (Supplementary Table 1). Strikingly, 5 diverse *P. aeruginosa* genomes were found to carry these genes arrayed and strictly conserved (Fig. 2b). The second gene in this cluster may regulate this element as it is predicted to encode a sodium-dependent phosphate transporter (PHO-4) known to be activated by iron limitation in the archaeon *Pyrococcus furiosus* (Zhu *et al.* 2013). The overall profile of this MGE warrants characterizing its role in the biology of *P. aeruginosa* M2.

To sum, our recent report demonstrated that, while displaying similar lethality to PAO1 and PAO10 strains in unburned mice; *P. aeruginosa* M2 has an LD_50_ 6-logs lower than PAO1 and PAO10 in burned mice yet a LD_50_ similar to PA14, a strain with comparable pathogenesis in seroma layers of thermal injuries (Brammer *et al.* 2021). This prompted sequencing this strain’s genome and using phylogenomics approaches to accentuate its unique characteristics or those shared with PA14. Dozens of genes were identified by this approach, with diverse functions like degradation of toxic aromatic compounds, iron scavenging, swarming motility and biofilm formation, defense against invasive DNA, and host assault. While the majority of probable LGTs are common to the *P. aeruginosa* accessory genome, a few instances of predicted LGT with divergent microbes illuminates novel MGEs that are heretofore uncharacterized for roles in *P. aeruginosa* biology. Our collective analysis, which entails probing genotype for observed phenotypic differences and similarities between *P. aeruginosa* strains, provides a rich resource for future assessment of the severity of disease in *P. aeruginosa* -infected burn patients.

## Data availability

The data underlying this article are available in the NCBI GenBank Database at ncbi.nlm.nih.gov/and can be accessed with PRJNA816887 (Bioproject ID for the PA M2 genome sequence) and with SRX14474414 (total reads in sequence read archive).

Supplemental material is available at *G3* online.

## Supplementary Material

jkac073_Supplementary_Figure_1

jkac073_Supplementary_Figure_2

jkac073_Supplementary_Material

jkac073_Supplementary_Table_S1
