# Supplementary figures and images for: Genome sequencing of Pseudomonas aeruginosa strain M2 illuminates traits of an opportunistic pathogen of burn wounds
Source: G3 (Bethesda). 2022 Mar 28;12(5):jkac073. doi: 10.1093/g3journal/jkac073 (PMC9073672; doi:10.1093/g3journal/jkac073)

FIG. S1

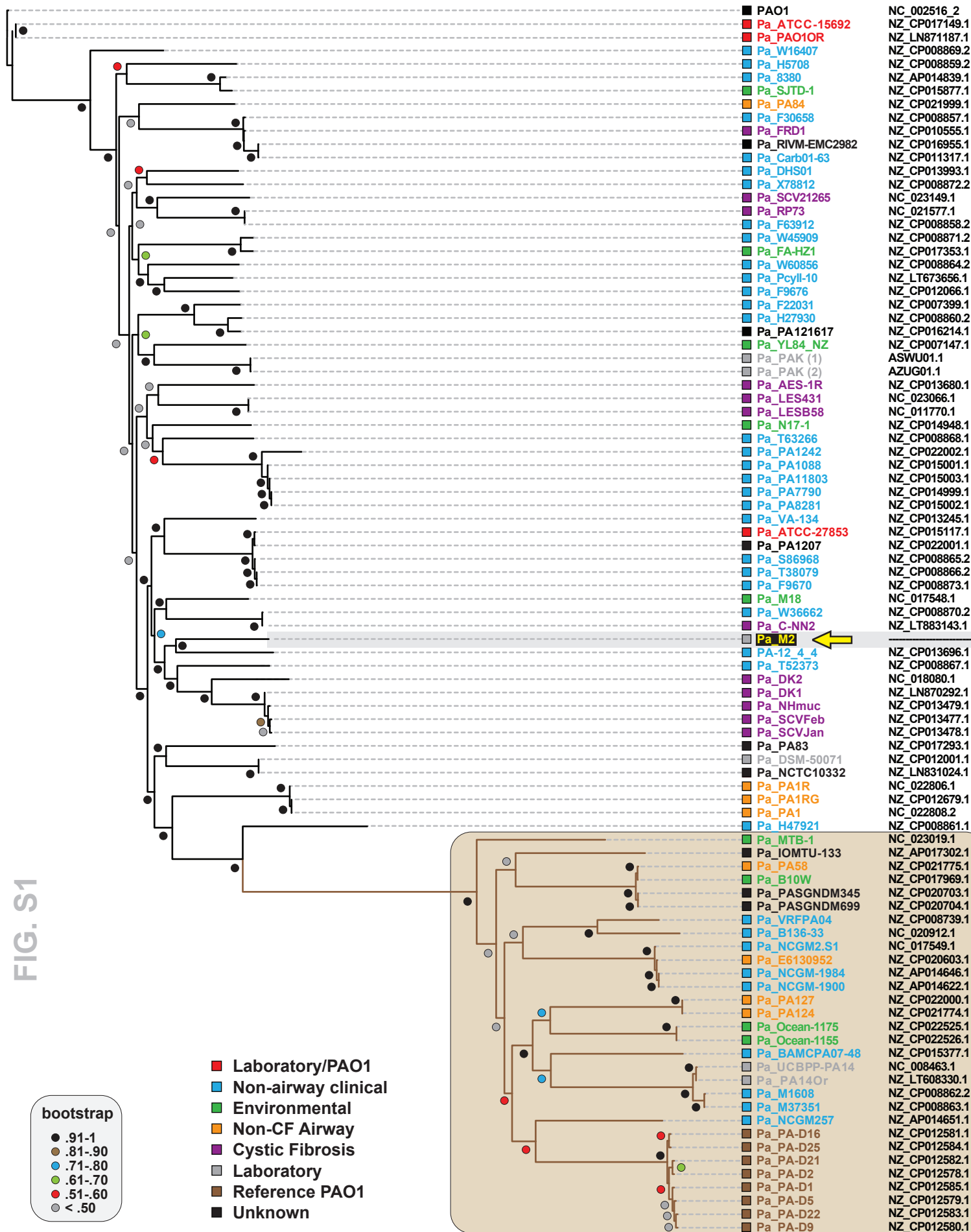

Supplement: jkac073_Supplementary_Figure_1 [file jkac073_supplementary_figure_1.pdf]
