# Supplementary material for: Genome sequencing of Pseudomonas aeruginosa strain M2 illuminates traits of an opportunistic pathogen of burn wounds
Source: G3 (Bethesda). 2022 Mar 28;12(5):jkac073. doi: 10.1093/g3journal/jkac073 (PMC9073672; doi:10.1093/g3journal/jkac073)
Supplement: jkac073_Supplementary_Figure_2 [file jkac073_supplementary_figure_2.pdf]

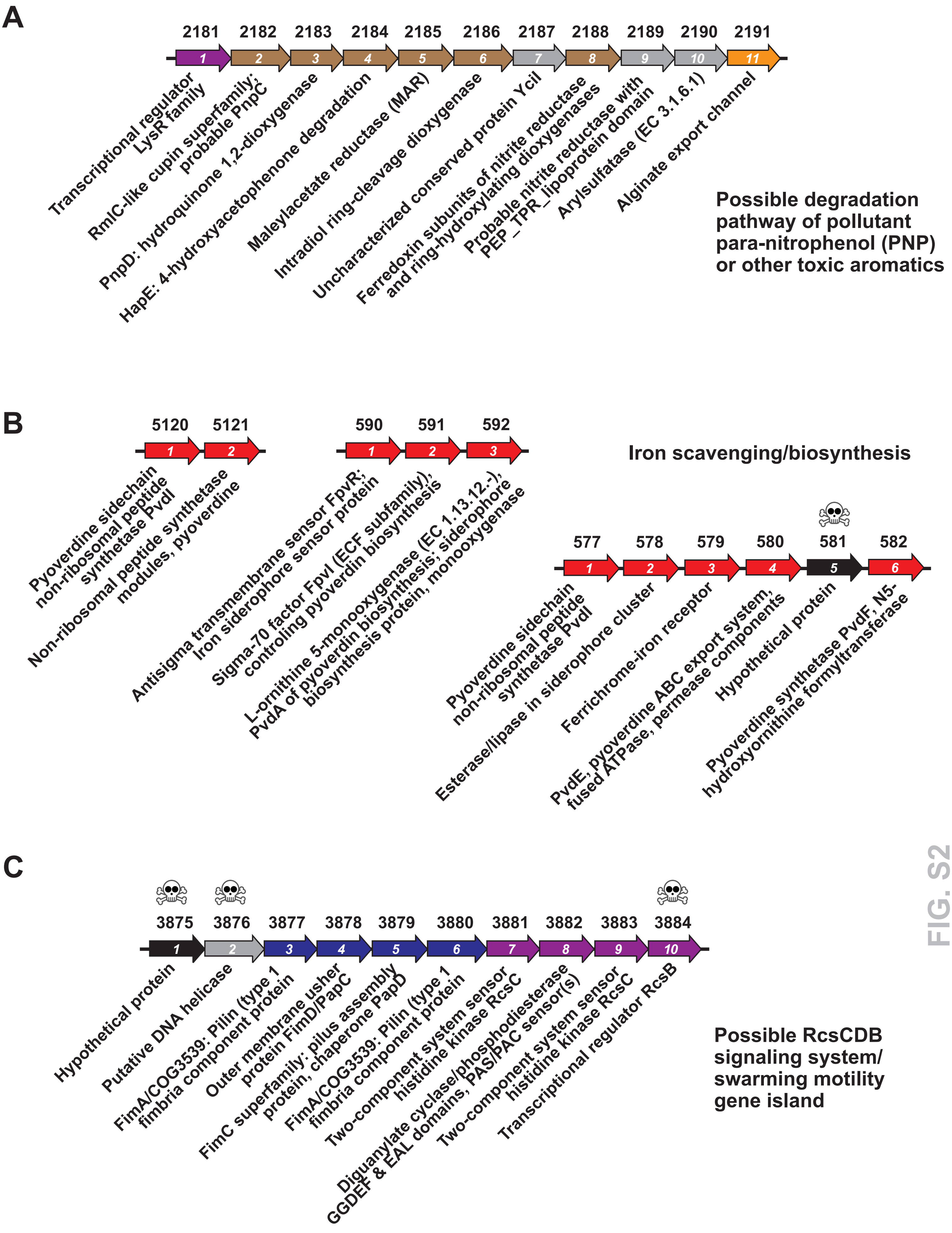
A
2181 2182 2183 2184 2185 2186 2187 2188 2189 2190 2191
1 2 3 4 5 6 7 8 9 10 11
Transcriptional regulator LysR family
RmlC-like cupin superfamily; probable PnpC
PnpD: hydroquinone 1,2-dioxygenase
HapE: 4-hydroxyacetophenone degradation
Maleylacetate reductase (MAR)
Intradiol ring-cleavage dioxygenase
Uncharacterized conserved protein YciI
Ferredoxin subunits of nitrite reductase and ring-hydroxylating dioxygenases
Probable nitrite reductase with PEP_TPR_lipoprotein domain
Arylsulfatase (EC 3.1.6.1)
Alginate export channel
Possible degradation pathway of pollutant para-nitrophenol (PNP) or other toxic aromatics
B
5120 5121
1 2
Pyoverdine sidechain non-ribosomal peptide synthetase PvdI
Non-ribosomal peptide synthetase modules, pyoverdine
590 591 592
1 2 3
Antisigma transmembrane sensor FpvR; Iron siderophore sensor protein
Sigma-70 factor FpvI (ECF subfamily), controling pyoverdin biosynthesis
L-ornithine 5-monooxygenase (EC 1.13.12.-), PvdA of pyoverdin biosynthesis; siderophore biosynthesis protein, monooxygenase
Iron scavenging/biosynthesis
577 578 579 580 581 582
1 2 3 4 5 6
Pyoverdine sidechain non-ribosomal peptide synthetase PvdI
Esterase/lipase in siderophore cluster
Ferrichrome-iron receptor
PvdE, pyoverdine ABC export system, fused ATPase, permease components
Hypothetical protein
Pyoverdine synthetase PvdF, N5-hydroxyornithine formyltransferase
C
3875 3876 3877 3878 3879 3880 3881 3882 3883 3884
1 2 3 4 5 6 7 8 9 10
Hypothetical protein
Putative DNA helicase
FimA/COG3539: Pilin (type 1 fimbria component protein
Outer membrane usher protein FimD/PapC
FimC superfamily: pilus assembly protein, chaperone PapD
FimA/COG3539: Pilin (type 1 fimbria component protein
Two-component system sensor histidine kinase RcsC
Diguanylate cyclase/phosphodiesterase GGDEF & EAL domains, PAS/PAC sensor(s)
Two-component system sensor histidine kinase RcsC
Transcriptional regulator RcsB
Possible RcsCDB signaling system/ swarming motility gene island

FIG. S2

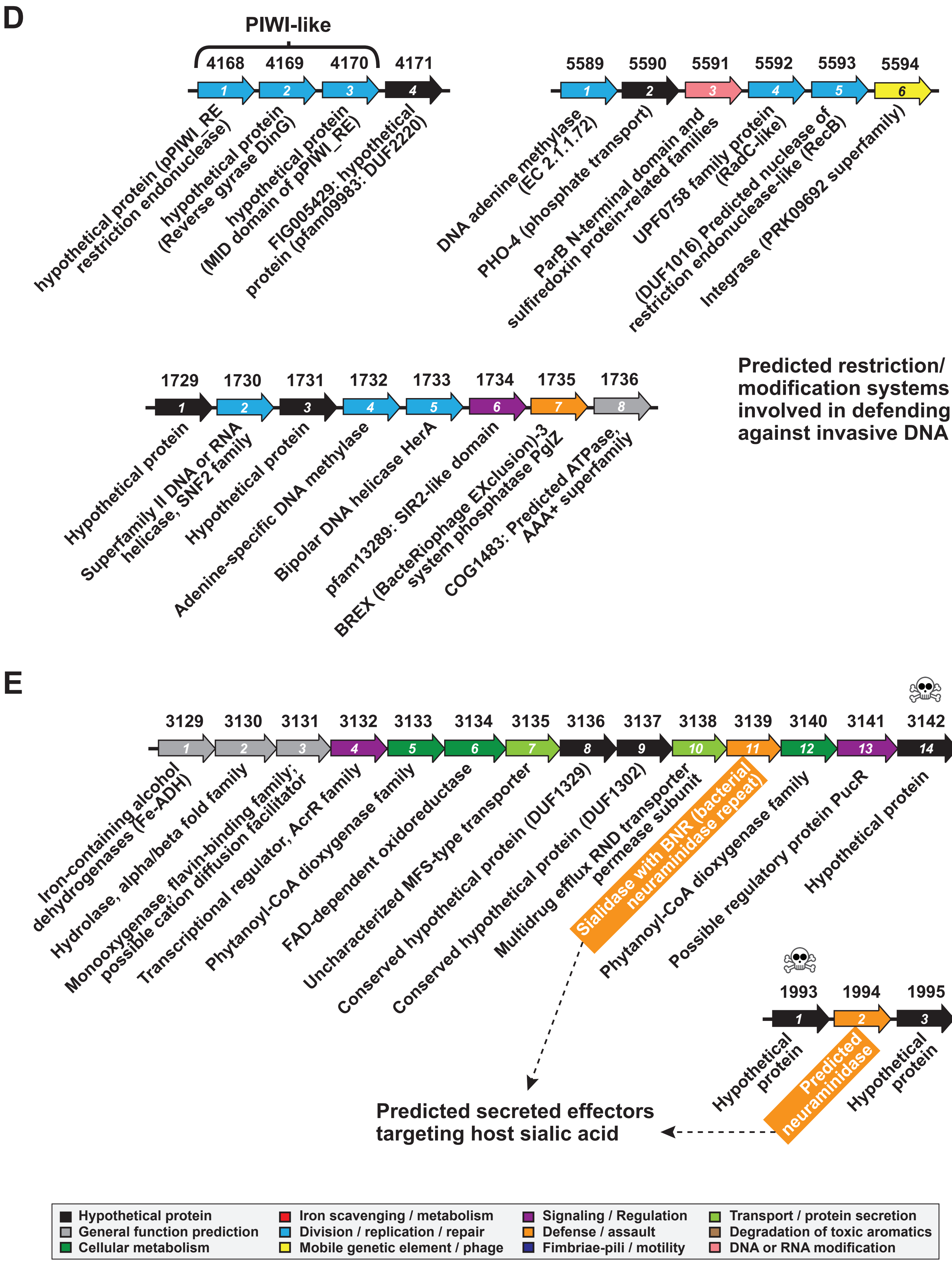
D
PIWI-like
4168
4169
4170
4171
1
2
3
4
hypothetical protein (pPIWI_RE restriction endonuclease)
hypothetical protein (Reverse gyrase DinG)
hypothetical protein (MID domain of pPIWI_RE)
FIG005429: hypothetical protein (pfam09983: DUF2220)
5589
5590
5591
5592
5593
5594
1
2
3
4
5
6
DNA adenine methylase (EC 2.1.1.72)
PHO-4 (phosphate transport)
ParB N-terminal domain and sulfiredoxin protein-related families
UPF0758 family protein (RadC-like)
(DUF1016) Predicted nuclease of restriction endonuclease-like (RecB)
Integrase (PRK09692 superfamily)
1729
1730
1731
1732
1733
1734
1735
1736
1
2
3
4
5
6
7
8
Hypothetical protein
Superfamily II DNA or RNA helicase, SNF2 family
Hypothetical protein
Adenine-specific DNA methylase
Bipolar DNA helicase HerA
pfam13289: SIR2-like domain
BREX (BacteRiophage EXclusion)-3 system phosphatase PglZ
COG1483: Predicted ATPase, AAA+ superfamily
Predicted restriction/ modification systems involved in defending against invasive DNA
E
3129
3130
3131
3132
3133
3134
3135
3136
3137
3138
3139
3140
3141
3142
1
2
3
4
5
6
7
8
9
10
11
12
13
14
Iron-containing alcohol dehydrogenases (Fe-ADH)
Hydrolase, alpha/beta fold family
Monooxygenase, flavin-binding family; possible cation diffusion facilitator
Transcriptional regulator, AcrR family
Phytanoyl-CoA dioxygenase family
FAD-dependent oxidoreductase
Uncharacterized MFS-type transporter
Conserved hypothetical protein (DUF1329)
Conserved hypothetical protein (DUF1302)
Multidrug efflux RND transporter permease subunit
Sialidase with BNR (bacterial neuraminidase repeat)
Phytanoyl-CoA dioxygenase family
Possible regulatory protein PucR
Hypothetical protein
1993
1994
1995
1
2
3
Hypothetical protein
Predicted neuraminidase
Hypothetical protein
Predicted secreted effectors targeting host sialic acid
Hypothetical protein
General function prediction
Cellular metabolism
Iron scavenging / metabolism
Division / replication / repair
Mobile genetic element / phage
Signaling / Regulation
Defense / assault
Fimbriae-pili / motility
Transport / protein secretion
Degradation of toxic aromatics
DNA or RNA modification

FIG. S2
