# Supplementary material for: Genome sequencing of Pseudomonas aeruginosa strain M2 illuminates traits of an opportunistic pathogen of burn wounds
Source: G3 (Bethesda). 2022 Mar 28;12(5):jkac073. doi: 10.1093/g3journal/jkac073 (PMC9073672; doi:10.1093/g3journal/jkac073)
Supplement: jkac073_Supplementary_Material [file jkac073_supplementary_material.docx]

**Supplemental Data**

**Figure S1.** Phylogeny estimation for PA M2 and 91 other PA strains. Tree is based on SNP divergence and computed using NASP (Sahl *et al.* 2016). Branch support is from 1000 bootstrap pseudoreplications. PA M2 is noted with the arrow. Brown shading, divergent clade. Asterisks denote strains PAO1 and PA14. NCBI accession numbers are listed for all genomes.

**Figure S2.** PA M2-defining genes clustered on contigs with functional enrichment and possible virulence factors. Clusters are noted for predicted functions in: (*A*) degradation of toxic aromatic compounds; (*B*) iron scavenging and biosynthesis, (*C*) swarming motility and biofilm formation; (*D*) RM systems defending against invasive DNA; (*E*) secreted effectors targeting host sialic acid.

**Table S1.** Information for proteins from LS-BSR and HaloBLAST analyses.
